# International Congress of Pharmacy

**Published:** 1910-09-17

**Authors:** 


### INTERNATIONAL CONGRESS OF PHARMACY.

The tenth International Congress of Pharmacy, which was held at Brussels from September 1 to 6, was attended by nearly 600 pharmacists of various nationalities, many of whom were official delegates from foreign countries. In all, twenty-one Governments were represented and a large number of Belgian and foreign pharmaceutical societies. The Pharmaceutical Society of Great Britain sent as delegates Mr. Edmund White, a member of the Society's Council, and Mr. E. S. Peck, of Cambridge. The main purpose of the meeting was to consider various questions of international pharmaceutical interest, but some of the matters brought forward were not of special importance so far as this country is concerned. The decision to form an International Pharmaceutical Association was perhaps the most useful piece of work performed by the Congress, for by means of such an organisation the ground can be prepared for future congresses, and questions which are not of real international importance can be eliminated from the programme. The principal subject brought forward was closely related to the work which has been done by the International Congress for the Unification of Potent Drugs. In an extremely interesting discussion it was demonstrated that the work of international unification could not be complete unless uniform methods of standardising drugs and galenical preparations were adopted, for the same preparation standardised by two different processes shows different results. The Congress therefore decided to ask the Belgian Government to convene an international conference for the purpose of deciding upon uniform methods for the analysis of potent drugs. It was also resolved to recommend the various authorities responsible for the production of pharmacopœias to adopt analytical reagents of a uniform standard. The question of the approximation of pharmacopœias is of even greater importance to medical practitioners than to pharmacists, and any work which tends in the direction of making the realisation of this ideal practicable will be heartily welcomed. Another subject discussed dealt with pharmacopœial revision, and the Congress approved of the suggestion that practising pharmacists should have a fair share in the work of compiling pharmacopœias. The best-attended meeting during the Congress was that at which the question of the sale of proprietary remedies was discussed, nearly 200 pharmacists of various nationalities being present. One might have expected that the discussion would have developed into a consideration of the means of checking the sale of these articles, but this was not the case. Although a number of speakers expressed disapproval of the traffic in proprietary remedies, the general opinion seemed to be that they were too well established to allow of any possibility of their sale being prevented, and that under the circumstances it was desirable to devise some scheme whereby the retailer could be assured of a profit on their sale. The matter is one of trade interest only, but the importance which attached to the question tends to indicate that proprietary medicines are as great an evil in other countries as in our own. A discussion also took place regarding the sale of proprietary antiseptic products and disinfectants, but this developed on different lines. The question here was not one of profit, and the opinion of the meeting was that in order to protect the public the sale of these products should be placed under official control. It was unanimously resolved by the Congress to recommend that all proprietary disinfectants should be tested both chemically and bacteriologically before their sale is permitted, and that the bactericidal strength of the disinfectant should be stated on the label. If these recommendations were carried into effect a very useful purpose would be achieved; but no doubt, in our own country at any rate, any attempt so to regulate the sale of proprietary disinfectants would be met by serious opposition from parties whose interests do not coincide with the public welfare.

A useful paper was read by the Chief Inspector of Belgian Pharmacies, who recommended that, wherever possible, pharmacists should make their own galenical preparations; but it was pointed out in the course of the discussion that many products could be much more satisfactorily prepared in manufacturing laboratories where special apparatus is installed. In a paper on the "Limitation of Pharmacies" it was shown that in those countries where the number of pharmacies is limited by law the position of the pharmacist is much better than in those countries where there is unlimited competition. The Congress approved of the principle of limitation, but this is merely an expression of opinion which cannot be expected to carry any weight, at any rate in Great Britain. In addition to these discussions several papers of a less general interest were contributed, one of which dealt with the assay of pepsin, a proposal being put forward for a definite international standard; it was decided to remit the question to a committee. In many respects the Congress was a distinct success, and not the least useful purpose served was that of bringing together in social intercourse pharmacists of more than twenty nationalities.

